# A comprehensive model for predicting the development of defense system of *Capparis spinosa* L.: a novel approach to assess the physiological indices

**DOI:** 10.1038/s41598-023-39683-5

**Published:** 2023-07-31

**Authors:** Sayed Fakhreddin Afzali, Hossein Sadeghi, Azin Taban

**Affiliations:** 1 Department of Natural Resources and Environmental Engineering, School of Agriculture, Shiraz University, Shiraz, 71441-65186 Iran; 2 Department of Horticultural Sciences, School of Agriculture, Shiraz University, Shiraz, Iran

**Keywords:** Physiology, Plant sciences

## Abstract

*Capparis* *spinosa* L. (caper) is a halophytic plant that grows in semi-arid or arid environments. The current study used an integrated experimental and computational approach to investigate the network of inter-correlated effective variables on the activity of antioxidant enzymes, proline, and photosynthetic pigments in stressed caper. To investigate the possible relationships among intercorrelated variables and understand the possible mechanisms, predictive regression modelling, principal component analysis (PCA), Pearson's correlation, and path analysis were implemented. PCA successfully discerned different salt ratio- and drought-specific effects in data in the current study, and treatments with higher growth indices are easily recognizable. Different salt ratios did not have a significant effect on the activity of four antioxidant enzymes, proline and photosynthesis pigments content of caper. While at the mean level, the activity of four antioxidant enzymes of SOD, POD, CAT, and APX significantly increased under drought stress by 54.0%, 71.2%, 79.4%, and 117.6%, respectively, compared to 100% FC. The drought stress also significantly increased the content of carotemoid (29.3%) and proline (by 117.7%). Predictive equation models with highly significant R^2^ were developed for the estimation of antioxidant enzyme activity and proline content (> 0.94) as well as pigments (> 0.58) were developed. Path analysis studies revealed that proline is the most important regressor in four antioxidant enzyme activities, while leaf tissue density was the most effective variable in the case of chlorophylls. Furthermore, the network of intercorrelated variables demonstrated a close relationship between caper's antioxidant defence system, pigments, and morphological parameters under stress conditions. The findings of this study will be a useful guide to caper producers as well as plant ecophysiological researchers.

## Introduction

Caper (*Capparis spinosa* L.), as a well-adapted plant in arid or semi-arid contexts as well as salty areas, can be used for increasing greenery and income in regions with extreme weather^1,2^. Soil erosion is a concern in these weather conditions. This plant has a deep root system that can resist drought and temperatures above 40 °C^3^. It also provides a vegetative canopy over soil surfaces and retains soil water in place^4^. It has been proposed that caper's salt tolerance mechanism is the localization of toxic ions in the shoot rather than the root and leaf, which are key sites of vital physiological and biochemical processes such as water and mineral absorption and photosynthesis, respectively^1^. Caper is a low-input crop that grows successfully in saline regions with less than 200 mm of annual rainfall without irrigation, where production of other commercial crops is impossible^1^. Caper is a low-input crop that grows successfully in saline regions with less than 200 mm of annual rainfall without irrigation, where production of other commercial crops is impossible^1,2,5^. Besides caper’s edible bud, flower, and fruits, it is also used for medicinal and culinary purposes^3^.

Some studies have attempted to reduce the effects of water deficit or salt stress through crop management and chemical treatment practices such as the use of CaCl_2_^6,7^. The addition of Ca^2+^ into the salted media or irrigation water leads to the acceleration of salt-induced growth inhibition in glycophytic plants^8^. Furthermore, Ca^2+^ sustains K^+^/Na^+^ selectivity and K^+^ transport in the Na^+^-challenged plants. Some studies have reported that the interaction between Na^+^ and Ca^2+^ influences ion relations and plant growth^9–11^. However, rare researchers have evaluated the stress effect of CaCl_2_ salt on plants comparing NaCl^12^. To cope with salt stress, plants have evolved many tolerance mechanisms mostly involved developing antioxidant defense system^13^. In a study on the xero-halophyte *Salsola drummondii* variations in growth, photosynthesis, antioxidant defense, water, and ion relations were examined. The resistance of *S. drummondii* in stressed conditions was related to plant interactions with soil and atmosphere. Precipitation, soil moisture, and electrical conductivity were all well correlated with leaf succulence, photosynthesis, water usage efficiency, compatible solutes, and antioxidant enzyme activities. During the monsoon period, with low vapor pressure deficit and moderate temperature, photosynthesis and stomatal conductance increased^14^. The efficient antioxidant mechanisms in halophytes can prepare plants to deal with subsequent stress factors by inducing a signal that activates the stress response and suppresses the oxidative burst within a short period of time following the stimulus action. The interrelationship of ROS scavengers results in spatiotemporal modulation of the ROS signaling network. In some cases, when salinity increased, glutathione peroxidase and glutathione reductase activity increased gradually, whereas catalase activity remained stable^15^.

Water deficit or drought stress, as well as salt stress, are two of the most common abiotic stresses that can significantly decrease photosynthesis. Furthermore, under stress conditions, a decrease in photosynthetic pigments and photosynthesizing tissue leads to a decrease in total growth^4,16^. Further, the production of reactive oxygen species (ROS) such as hydrogen peroxide (H_2_O_2_), hydroxyl radical (OH^-^), superoxide radical (O_2_^-^), and singlet oxygen (O_1_^-^) increases under stress conditions which leads to the enhancement of enzymatic and non-enzymatic antioxidant^17^. It is argued that accumulating the higher concentrations of osmotic solutes and greater activity of antioxidant enzymes in plants can increase the plant's adaptability to stress conditions^18^. A large number of studies have focused on the higher activity of the antioxidant defense system which occurred under environmental stresses^17,19–21^. The metabolic disorders caused by the content of deteriorated moisture in the soil or disproportionate accumulation of Na^+^ and Cl^−^ in plants lead to salt stress^22^. Based on the results of previous studies^1,4,23^, salt stress affects the content of moisture, proline, and glycine betaine in plants, and the activation of anti-oxidative enzymes such as catalase (CAT), peroxidase (POD), superoxide dismutase (SOD) and ascorbate peroxidase (APX). As a result, the content of enzymatic and non-enzymatic antioxidant and photosynthetic pigments is an important biochemical indicator for evaluating a plant's stress tolerance, despite the fact that only one study looked at the possible link and mechanism between the antioxidant defense system and the pigments content of stressed plants^24^. Statistical techniques such as principal component analysis (PCA), correlation coefficient, stepwise regression modelling, and path analysis are currently very useful in determining possible relationships between inter-correlated variables and understanding the possible morphophysiological and biochemical mechanisms of plant tolerance to salinity stress^1,18,21,23^.

In brief, this study was designed due to a lack of knowledge on the mechanism, important effective variables, and the possible relationship between antioxidant defense system and photosynthetic pigments under stress conditions, as well as the importance of caper for the environment and industry. This is the first attempt, to the best of the author's knowledge, to develop predictive models for the estimation of proline, antioxidant enzyme activity, and photosynthetic pigments under drought and salt stress. Furthermore, predicting the reaction of different plants under these conditions is extremely valuable and can be applied to other plants as well as the advancement of research in this field.

In light of the above, the main objectives of this study were to 1—Evaluating and comparing the effect of different salt ratios of calcium chloride to sodium chloride (1:0, 1:1, 1:3, and 1:5) on antioxidant defense system, photosynthesis pigments, and morphophysiological parameters of caper under drought stress (75 and 100% FC), 2—Exploring the possible correlation between measured parameters, 3—Quantifying the direct and indirect effect of different variables on proline, antioxidant enzymes activity, and photosynthesis pigments through the path analysis, and 4—Developing predictive models for the estimation of proline, antioxidant enzyme activity, and photosynthesis pigments under drought and salt stress using stepwise regression modeling. As a novelty, there had previously been no caper physiological and biochemical modelling under stressed conditions.

## Materials and methods

### Plant material

Caper seeds were collected from Farashband (28°52′17″ N 52°05′30″ E), Fars Province, Iran. The (Iranian Government Organization) Natural Resources and Watershed Management Organization granted authorization to collect seeds. Following the separation of healthy and uniform seeds, they were rinsed with deionized water and sterilized for 2 min with ethanol (95%). After breaking the dormancy with sulfuric acid (Merck, Germany, 98%-10 min), they were rinsed several times with running water before being placed in Petri dishes containing 5 mL deionized water and Whatman filter paper. For 4 weeks, the Petri dishes were kept in a growth chamber (1300 STC Mod, NoorSanat-Ferdows Company, Karaj, Iran) at 26 °C (day) and 22 °C (night), 4000 lx, and 16 h photoperiod. After germination, the seeds were transplanted into the 4 L plastic pots containing silty loam soil. Some of the important soil characteristics are summarized in Table S1 of supplementary data. The pots were kept out of the greenhouse (outdoors) in 2015 from April to September.

### Ethical approval

Experimental research and greenhouse studies on Caper (*Capparis spinosa* L.), including the collection of plant material, complied with relevant institutional, national, and international guidelines and legislation.

### Experimental procedure

Drought stress consisted of two levels of 100 and 75% FC. Furthermore, the levels of drought treatment were applied by considering the weighting method^25^. Every day, after weighing, water was added to the pots to indirectly determine the amount of moisture in the soil and, thus, to preserve the specifications of each treatment. The weight of the pots gradually decreased throughout the course of the observations as the plants used water and evaporation occurred^5^. Finally, four different salt ratios of calcium chloride to sodium chloride (Merck, Germany) (1:0, 1:1, 1:3, and 1:5) were utilized. The 70 days old seedlings of capers were subjected to salt stress by saline water containing different salt ratios of CaCl_2_:NaCl. To avoid the salinity shock, the NaCl content was gradually increased over the course of two weeks. The electrical conductivity (EC) of the various salt ratio treatments was held constant at 8 dS/m. Seedlings were kept close to field capacity during the first growth phase. The drought and salinity conditions were administered to the pots-based weighing method after 70 days^4^.

### Measurement

Tissue density including leaf density and root density was measured using the formula (1) based on the method of Zhang et al.^26^:

1 $$\mathrm{Tissue }\left(\mathrm{leaf or root}\right)\mathrm{density}=\frac{Dry mass of leaf or root }{Turgid mass of leaf or root}$$

The calculation of turgid mass was extensively discussed by Zhang et al. The salt sensitivity index (SSI) of plants in different treatments was calculated using the below formula (2) by Sezer et al.^27^:

2 $$\mathrm{SSI}=\left(\frac{ABF \left(Salinity\right)-ABF \left(control\right)}{ABF (control)}\right)\times 100$$

where; ABF (salinity) is the aerial fresh biomass value for salinity treatments, and ABF (control) is the aerial fresh biomass for the control treatment.

Other parameters such as shoot growth during stress (SGS), number of new leaf growth during stress (NLGS), and increasing the length of the largest leaf during stress (ILLS) were measured at two times: (i) before imposing drought and salinity and (ii) at the end of the trial.

### Pigments content

The content of chlorophyll a and b, total chlorophyll, and carotenoid in the samples were calculated through the method provided by Arnon and Whatley^28^. Briefly, A 500 mg fresh leaf sample was dissected into small pieces, and pigments were extracted in 80% cold acetone (Merck, Germany). The test tubes were incubated at room temperature in the dark for 12 h. After decanting the supernatant, 3 mL of cold acetone was added to the residue, which was then incubated at room temperature for 30 min. Following supernatant pooling, the volume was raised to 10 mL and cold acetone was added. Carotenoid and chl extracts were placed in a cuvette, and their absorption was determined with a spectrophotometer (Model Epoch Biotek, Winooski, VT, USA) at 645, 663, and 470 nm. Then, the content of the pigments was computed by using the standard formulas of^29^.

### Proline concentration

The free proline content of the leaves was extracted and measured using Ghanaatiyan and Sadeghi^19^ procedure using sulpho-salicylic acid and acid ninhydrin (Merck-Germany). In microtubes, 0.1 g of fresh leaf sample was mixed with 1 ml ethanol and incubated for 12 h at room temperature. Following that, 100 μL of leaf extract was mixed with 900 μL of distilled water and 500 μL of ninhydrin solution (including ninhydrin, acetic acid glacial and phosphoric acid 6 M). They were then incubated for 45 min in a 65 °C oven. A spectrophotometer was used to measure the absorbance of the samples at 520 nm (Model Epoch Biotek, Winooski, VT, USA). Finally, the concentration of proline was expressed as μmol g^-1^ fresh weight^19^.

### Antioxidant enzyme activity

The activity of SOD was measured using nitro blue tetrazolium (NBT) by measuring the absorbance at 560 nm^16^. A reaction mixture (3 mL) contained 50 mM sodium phosphate buffer (pH 7.8), 13 mM methionine, 75 μM NBT, 10 μM EDTA, 2 mM riboflavin, and 100 μL enzyme extract. One unit of SOD activity is the amount of enzyme that inhibits the photochemical degradation of NBT by 50%.

In addition, POD activity was assessed based on the method of Fakhari and Sadeghi^17^. The reaction mixture (0.2 mL) comprised 50 mM guaiacol, 50 mM phosphate buffer (pH 7.8), and 2% H_2_O_2_. The absorbance was read at 436 nm for 5 min. An absorbance change of 0.01 units per minute was considered one unit of POD activity.

Further, the disappearance of H_2_O_2_ at 240 nm (ε = 40 mM cm^−1^) was monitored for determining the activity of CAT. To measure this, the solution (3 mL) contained 0.1 mL enzyme extract, 0.1 M H_2_O_2_, and 50 mM phosphate buffer (pH 7.8). A reduction in H_2_O_2_ prompted a decrease in optical density at 240 nm. An absorbance change of 0.01 units per minute was regarded as one unit of CAT activity^21^.

Finally, ascorbate peroxidase (APX) activity was measured by mixing 50 mM Na-phosphate buffer (pH = 6), 0.1 mM H_2_O_2_, 0.1 mM EDTA, and 0.5 mM ascorbate, as well as 0.2 mL of enzyme extract, and reading light absorption at 290 nm^19^.

### Statistical analysis

This study was conducted as a factorial experiment with two factors and three replications using a completely randomized (CRD). The first factor was CaCl_2_:NaCl salt ratios of 1:0, 1:1, 1:3, and 1:5, whereas the second factor was drought stress at two levels (100 and 75% FC). The hypothesis of the data normal distribution was tested in SAS 9.3 software by running a univariate normality test on residuals in the analysis of variance (ANOVA) model for all of the traits under study. Furthermore, the main impact of the factors, as well as their interactions, were evaluated using ANOVA and SAS's general linear model (GLM) procedure. Finally, the mean of the main treatment factors and their interactions was compared through Duncan’s multiple range test at *p* ≤ 0.05. All regression modeling studies as well as performing path analysis were performed in SPSS 21.0 (IBM Inc., USA) software package. The stepwise multiple regression techniques were employed to determine the most effective variables which have a significant effect (*p* ˂0.05) on antioxidant enzymes, proline, and photosynthetic pigment (as important stress indicators).

3 $$\gamma = \beta_{0} + \beta_{{1}} {\text{x}}_{{1}} + \cdots + \beta_{{\text{k}}} {\text{x}}_{{\text{k}}}$$

where *γ* is the dependent variable (including different antioxidant enzymes, pigments, and the content of proline, x_1_, x_k_ are the predictor variables and β_j_ 's are the regression confidence (Kumar et al.). The measured variables from the previous study^30^ were also used to construct the regression models and path diagram (Those variables are listed in Table S3 of supplementary data). Statgraphics software (Version 19) produced the Pearson correlation (correlation plot), and MINITAB software was used to perform principal component analysis (PCA) (Version 19).

## Results

### Morphological parameters and indexes

The effects of drought and salinity stress (various salt ratios) on morphological parameters and indices of C. *spinosa* are shown in Table 1. According to the ANOVA table (Table S2 of supplemental data), using different salt ratios and the combined effect of salt × ratio drought stress had a significant effect (*p* < *0.01*) on the shoot/root ratio, although the effect of drought stress (75% FC) was not significant. The highest shoot/root ratio was obtained at 75% FC with a salt ratio of 1:0 (CaCl_2_:NaCl), while the lowest was obtained at 75% FC with a salt ratio of 1:5 (CaCl_2_:NaCl) and 75%FC with an 85.8% reduction compared to the ratio of 1:0. (CaCl_2_:NaCl). In terms of the main effect of salt ratios on shoot/root, increasing the concentration of NaCl from 1:0 to 1:5 (CaCl_2_:NaCl) significantly reduced shoot/root by 62.4%. (Table 1).

**Table 1:** The effects of drought and salinity stress (different salt ratio) on morphological parameters and indexes of *C. spinosa*.

Factors	FC (%)	Salt ratio (CaCl_2_:NaCl)				Mean
		01:00	01:01	01:03	01:05	
Shoot/Root	75%	2.11 ± 0.51 a	1.44 ± 0.51 bc	0.73 ± 0.07 de	0.38 ± 0.06 e	1.17 A
	100%	1.04 ± 0.17 cd	1.21 ± 0.37 cd	1.81 ± 0.17 ab	0.79 ± 0.01 de	1.21 A
	Mean	1.57 A	1.33 A	1.27 A	0.59 B	
Leaf tissue density	75%	0.22 ± 0.14 abc	0.12 ± 0.04 cd	0.12 ± 0.01 cd	0.06 ± 0.00 d	0.13 B
	100%	0.12 ± 0.01 cd	0.13 ± 0.02 bcd	0.25 ± 0.04 a	0.24 ± 0.07 ab	0.18 A
	Mean	0.17 A	0.13 A	0.18 A	0.15 A	
Root tissue density	75%	0.19 ± 0.05 ab	0.2 ± 0.00 ab	0.21 ± 0.04 ab	0.28 ± 0.01 a	0.22 A
	100%	0.23 ± 0.02 ab	0.16 ± 0.04 b	0.23 ± 0.01 ab	0.23 ± 0.08 ab	0.21 A
	Mean	0.21 AB	0.18 B	0.22 AB	0.25 A	
SSI	75%	0.00 ± 0.11 a	-0.11 ± 0.00 ab	-0.56 ± 0.00 c	-0.50 ± 0.06 c	-0.29 A
	100%	0.00 ± 0.33 a	-0.22 ± 0.11 abc	-0.22 ± 0.33 abc	-0.39 ± 0.17 bc	-0.21 A
	Mean	0.00 A	-0.170 AB	-0.390 BC	-0.440 C	
SGS (cm)	75%	10.67 ± 9.50 ab	7.00 ± 1.00 ab	7.00 ± 2.00 ab	4.50 ± 3.91 b	7.29 A
	100%	2.67 ± 1.53 b	3.67 ± 2.08 b	15.33 ± 6.66 a	3.67 ± 0.58 b	6.33 A
	Mean	6.67 AB	5.33 B	11.17 A	4.08 B	
NLGS	75%	1.00 ± 1.00 b	1.33 ± 1.53 b	4.00 ± 1.00 ab	4.00 ± 0.00 ab	2.58 A
	100%	1.67 ± 2.08 ab	1.00 ± 1.00 b	4.33 ± 1.53 a	3.00 ± 2.65 ab	2.50 A
	Mean	1.33 B	1.17 B	4.17 A	3.50 A	
ILLS (cm)	75%	1.17 ± 0.58 a	0.70 ± 0.50 ab	0.60 ± 0.26 ab	0.70 ± 0.00 ab	0.79 B
	100%	0.47 ± 0.25 b	0.40 ± 0.53 b	0.40 ± 0.17 b	0.30 ± 0.17 b	0.39 A
	Mean	0.82 A	0.55 A	0.50 A	0.50 A	

Means within each parameter followed by the same letter are not significantly different at *p* ≤ 0.05 using Duncan’s test. Capital letters are used for the main effects.

*SSI* salt sensitivity index, *SGS* shoot growth during stress, *NLGS* number of new leaves growth during stress, *ILLS* increasing the length of largest leaf during stress.

Drought stress showed a significant (*p* < *0.05*) effect on leaf tissue density (LTD), whereas different salt ratios had no meaningful effect (Table S2 of supplementary data). At the mean level, applying drought stress decreased leaf tissue density by 27.8% compared to 100% FC (Table 1).

Unlike drought stress, applying different salt ratios and the combined effect of salt ratio drought stress had no significant effect on root tissue density (RTD), with the highest RTD obtained at a ratio of 1:5 (CaCl_2_:NaCl) under 75% FC (Table S2 of supplementary data and Table 1).

The salt sensitivity index (SSI) determines a stressed plant's ability to maintain growth, development, and key physiological and biochemical processes in a very saline environment (Table S2 of supplementary data) Only the primary effect of salt ratio on SSI was significant (p < 0.01). According to Table 1, increasing the NaCl concentration from 1:0 to 1:5 (CaCl_2_:NaCl) raised SSI values by 44% due to lowering aerial fresh biomass (formula (2)). The 1:0 (CaCl_2_:NaCl) ratio was used as the control.

Only the combined effects of drought stress × different salt ratio had a significant effect (*p* < *0.05*) on shoot growth during stress (SGS) and the number of new leaves growth during stress (NLGS) according to the ANOVA table (Table S2 of supplementary data) (Table S2 of supplementary data). The highest SGS value was recorded at a salt ratio of 1:3 (CaCl_2_:NaCl) under 100% FC, with a more than fivefold increase compared to a salt ratio of 1:0 (CaCl_2_:NaCl) at the same drought level.

Different salt ratios had a significant (*p* < *0.05*) effect on NLGS (Table S2 of supplementary data). Similar to SGS, the highest NLGS was recorded at a ratio of 1:3 (CaCl_2_:NaCl) under 100% FC, with a more than 2.5-fold increase over the ratio of 1:0 (CaCl_2_:NaCl) at the same drought level.

Drought stress exhibited a significant (*p* < *0.05*) effect on the increasing length of the largest leaf during stress (ILLS) (Table S2 of supplementary data). According to Table 1, at the mean level, the size of the largest leaf increased by 102.5% at 75% FC compared to 100% FC (Table 1).

### Photosynthetic pigment content

According to the ANOVA table, drought stress and different salt ratios had no significant effect on photosynthetic pigments except for carotenoids, which were significantly (*p* < *0.01*) affected by drought stress (Table S2 of supplementary data).

Table 2 depicts the mean comparison for the pigments content of caper under different drought stress and various CaCl_2_ to NaCl ratios. Based on the obtained results, at the mean level, the content of chlorophyll *a*, chlorophyll *b*, total chlorophyll, and carotenoid (29.3%) increased under drought stress. The results of the mean comparison of pigments content in the different ratios of CaCl_2_ to NaCl indicated that at 75% FC the content of total chlorophyll, and chlorophyll *a*, increased up to 1:3 salt ratios by 24.6% and 17.6%, respectively (compared to the ratio of 1:0).

**Table 2:** The effects of drought and salinity stress (different salt ratio) on photosynthesis pigments of *C. spinosa*.

Factors	FC (%)	Salt ratio (CaCl_2_:NaCl)				
		01:00	01:01	01:03	01:05	Mean
ChlT (mg/gr Fw)	75%	13.91 ± 0.36 ab	14.61 ± 2.03 ab	17.33 ± 1.24 a	17.33 ± 1.73 a	15.79 A
	100%	15.54 ± 1.28 ab	16.01 ± 1.15 a	14.47 ± 0.48 ab	9.89 ± 7.93 b	13.98 A
	Mean	14.73 A	15.31 A	15.90 A	13.61 A	
Chl *a* (mg/grFw)	75%	10.8 ± 0.39 a	11.01 ± 1.44 a	12.7 ± 0.55 a	12.45 ± 0.86 a	11.74 A
	100%	11.89 ± 1.29 a	12.24 ± 1.55 a	10.05 ± 0.26 ab	6.65 ± 5.34 b	10.21 A
	Mean	11.34 A	11.62 A	11.38 A	9.55 A	
Chl *b* (mg/gr Fw)	75%	3.11 ± 0.21 a	3.61 ± 0.59 a	4.63 ± 0.73 a	4.87 ± 0.89 a	4.05 A
	100%	3.65 ± 0.10 a	3.76 ± 0.45 a	4.41 ± 0.38 a	3.24 ± 2.6 a	3.77 A
	Mean	3.38 A	3.68 A	4.52 A	4.06 A	
Car. (mg/gr Fw)	75%	4.52 ± 0.13 a	4.52 ± 0.13 a	4.38 ± 0.22 a	3.71 ± 0.57 ab	4.28 A
	100%	3.14 ± 0.37 ab	3.68 ± 0.32 ab	3.9 ± 0.14 ab	2.52 ± 2.02 b	3.31 B
	Mean	3.83 AB	4.10 AB	4.14 A	3.11 B	

Means within each parameter followed by the same letter are not significantly different at *p* ≤ 0.05 using Duncan’s test. Capital letters are used for the main effects.

*ChlT* total chlorophyll, *Chl a* chlorophyll *a*, *Chl b* chlorophyll *b*, *Car* carotenoid.

In contrast, increasing the NaCl concentration from 1:0 to 1:5 decreased the carotenoid content by 18.7% at the mean level (Table 2). The results indicate that the simultaneous application of NaCl and drought stress had a higher effect on the growth of photosynthetic pigments than either stress alone.

### Antioxidant enzymes activity

According to the ANOVA table, only drought stress had a significant (*p* < *0.01*) effect on four antioxidant enzymes activity, although the effect of varied salt ratios and the combined effect of drought stress × salt ratio was not significant (Table S2 of supplementary data). At the mean level, under drought stress, the activity of four antioxidant enzymes, SOD, POD, CAT, and APX, increased by 54.0%, 71.2%, 79.4%, and 117.6%, respectively, compared to 100% FC (Table 3). Also, the highest value of enzyme activity was reported in all four enzymes at a ratio of 1:3 (CaCl_2_: NaCl) under 75% FC (Table 3).

**Table 3:** The effects of drought and salinity stress (different salt ratio) on antioxidant enzymes activity of *C. spinosa*.

Factors	FC (%)	Ratio				
		01:00	01:01	01:03	01:05	Mean
SOD (u/mg protein)	75%	10.59 ± 0.75 abcd	11.92 ± 1.34 abc	14.03 ± 1.47 a	12.75 ± 4.61 ab	12.32A
	100%	7.42 ± 1.03 cd	8.73 ± 0.52 bcd	9.32 ± 0.46 abcd	6.53 ± 5.24 d	8.00 B
	Mean	9.01 A	10.33 A	11.67 A	9.64 A	
POD (u/mg protein)	75%	9.65 ± 2.87 abc	13.04 ± 0.45 a	13.4 ± 0.68 a	11.52 ± 3.90 ab	11.9 A
	100%	6.82 ± 0.58 c	7.5 ± 0.38 bc	7.91 ± 0.41 bc	5.56 ± 4.46 c	6.95 B
	Mean	8.23 A	10.27 A	10.66 A	8.54 A	
CAT (u/mg protein)	75%	8.251 ± 0.32 abc	9.24 ± 1.60 ab	11.27 ± 0.67 a	9.60 ± 3.69 ab	9.60 A
	100%	5.01 ± 0.27 c	5.27 ± 0.23 c	6.07 ± 1.22 bc	5.04 ± 4.04 c	5.35 B
	Mean	6.63 A	7.26 A	8.67 A	7.32 A	
APX (u/mg protein)	75%	6.62 ± 0.78 a	7.86 ± 0.86 a	8.85 ± 0.37 a	7.30 ± 2.89 a	7.66 A
	100%	3.70 ± 0.19 b	3.80 ± 0.10 b	3.83 ± 0.24 b	2.73 ± 2.19 b	3.52 B
	Mean	5.16 A	5.83 A	6.34 A	5.02 A	
Proline (µg/gr Fw)	75%	3.58 ± 0.78 ab	4.64 ± 0.39 a	5.05 ± 0.18 a	3.86 ± 2.12 ab	4.30 A
	100%	1.43 ± 0.44 c	2.03 ± 0.17 bc	2.44 ± 0.46 bc	2.01 ± 1.61 bc	1.98 B
	Mean	2.51 A	3.33 A	3.74 A	2.93 A	

Means within each parameter followed by the same letter are not significantly different at *p* ≤ 0.05 using Duncan’s test. Capital letters are used for the main effects.

*SOD* superoxide dismutase, *POD* peroxidase, *CAT* catalas, *APX* ascorbate peroxidase.

### Proline content

Drought stress exhibited a significant (*p* < *0.01*) effect on C. *spinosa* proline content (Table S2 of supplementary data). At the mean level, the proline content of C. *spinosa* increased significantly at 75% FC by 117.7% compared to 100% FC. Similar to antioxidant enzymes, the highest proline content was reported at the ratio of 1:3 (CaCl_2_:NaCl) under 75% FC (Table 3).

### Predictive model equations for antioxidant enzymes activity, proline, and photosynthetic pigments of C. spinosa

Multivariate linear stepwise regression was applied to (i) indicating the variables with the most effect on the activities of SOD, POD, CAT, and APX as well as the content of proline, ChlT, Chl *a*, Chl *b,* and carotenoid as the most important stress tolerance indices and (ii) develop the model for predicting antioxidant enzymes activities, proline and pigments content of *C. Spinosa* under drought and different salt ratio stress. Table 4 shows the predictive model equations in which γ is the dependent variable. The model validation was evaluated by using the coefficient of determination (*R*^*2*^), and ANOVA test, and comparing the predicted and measured dependent values. All regression relationships were performed, and only those that were significant were reported. The most effective independent variables on antioxidant enzymes, according to the stepwise regression, were proline content, ChlT, Chl *a*, chlorophyll index, shoot moisture, and root length. CAT activity was the most important indicator for predicting proline content of C. *spinosa* under stress conditions. The most effective variables for photosynthetic pigments were leaf tissue density, SGS, NLGS, shoot/root, and ChlT. The *R*^*2*^ values ranged from 0.893 to 0.998, indicating that the regression models had a high linear fitness (except for Chl *a*, 0.584). Furthermore, according to the ANOVA results, all of the predictive equations were statistically significant, with high F values and low *P* values (lower than 0.027) (Table 4).

**Table 4:** Predictive model equations with measure, predicted, R^2^ and ANOVA result values for antioxidant enzymes activity, proline and photosynthethic pigments of *C. spinosa*.

Dependent factor (γ)	Predictive Eq. (γ =)	γ_max_		R^2^	Adjusted R^2^	ANOVA	
		Measured	Predicted			*F value*	*Sig*
SOD (u/mg protein)	0.06 + 1.27 (proline content) + 0.46 (ChlT) – 0.03 (Chl index)	14.03	14.01	0.996	0.994	373.80***	0.000
POD (u/mg protein)	− 4.69 + 2.06 (proline content) + 0.10 (shoot moisture)	13.40	13.2	0.981	0.974	132.15***	0.000
CAT (u/mg protein)	1.93 + 1.77 (proline content)	11.27	10.87	0.945	0.936	103.35***	0.000
APX (u/mg protein)	0.74 + 1.43 (proline content) + 0.36 (Chla) – 0.15 (root length)	8.85	12.04	0.998	0.996	532.92***	0.000
Proline (µg/gr Fw)	0.86 + 0.53 (CAT activity)	5.05	6.83	0.945	0.936	103.35***	0.000
ChlT (mg/gr Fw)	18.65 – 38.58 (L. Density) + 0.34 (SGS)	17.33	16.4	0.833	0.766	12.46*	0.011
Chla (mg/gr Fw)	14.39 – 21.70 (L. Density)	12.70	11.78	0.584	0.515	8.44*	0.027
Chlb (mg/gr Fw)	3.77 + 0.36 (NLGS) – 4.89 (L. Density)	4.87	4.92	0.872	0.820	16.996**	0.006
Car. (mg/gr Fw)	0.27 + 0.18 (POD) + 0.74 (shoot/root) + 0.07 (ChlT)	4.52	4.54	0.984	0.972	83.463***	0.000

***, ** and * are significant at 0.001, 0.01 and 0.05 level of probability, respectively.

*SOD* superoxide dismutase, *POD* peroxidase, *CAT* catalas, *APX* ascorbate peroxidase, *ChlT* total chlorophyll, *Chl a* chlorophyll a, *Chl b* chlorophyll b, *Car* carotenoid.

### PCA analysis

**Figure 1:**
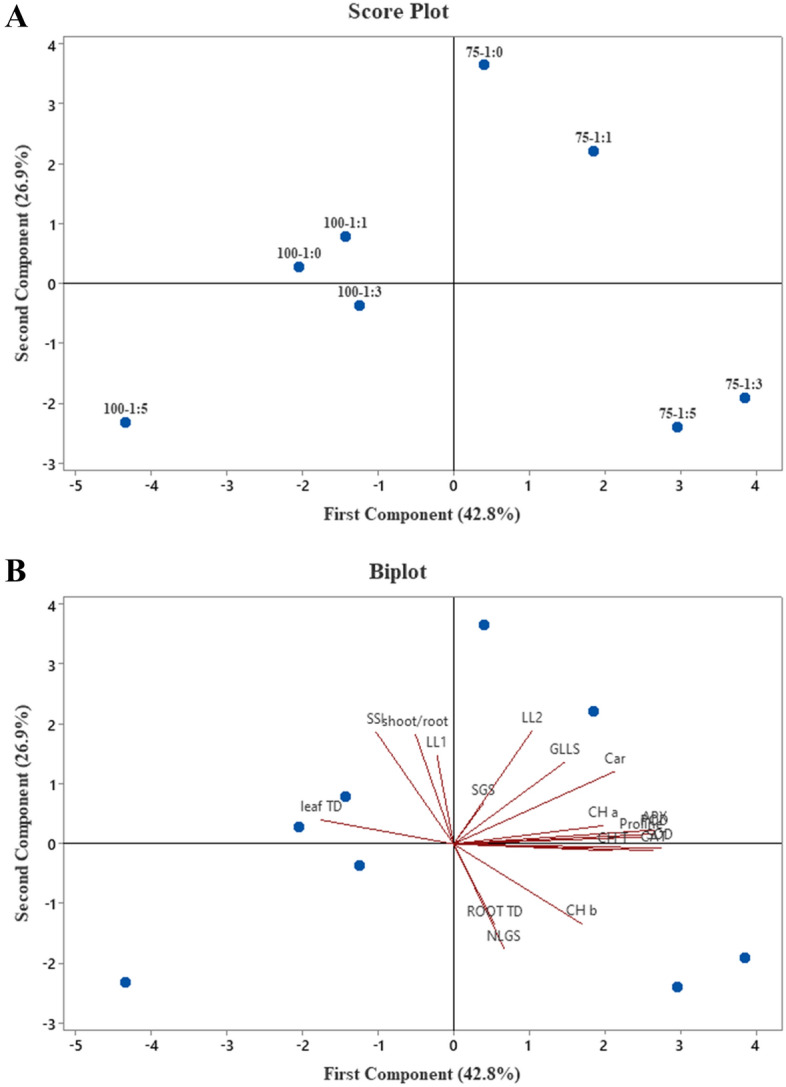
Principal components analysis (PCA) of drought stress-different salt ratio (CaCl_2_:NaCl) treatments including score plot (**A**) and biplot (**B**). *GLLS* growing largest leaf during stress, *SGS* shoot growing during stress, *NLGS* number of new leaves growth during stress, *LL1* length of largest leaf before salt and drought stress, *LL2* length of largest leaf at the end of trial, *Car* carotenoid, *SSI* salt sensitivity index, *TD* tissue density.

Principal components analysis (PCA) was used to create a better graphical representation of distinct drought stress-salt ratio treatments (Fig. 1). Figure 1A,B depicted the drought stress-different salt ratio (CaCl_2_:NaCl) score plot and biplot, respectively. The first two components were subjected to PCA analysis using 18 variables. The first and second components each accounted for 42.8% and 26.9% of the total variance (69.7%). Plants with 75% FC and salt ratios of 1:3 and 1:5 are mostly found on the lower right side of the score plot, while those with 75% FC and salt ratios of 1:0 and 1:1 are mostly found on the upper right side of the plot, and unstressed capers (100% FC) treated with different salt ratios (1:0 to 1:5) are mostly found on the left side of the score plot. Treatments with higher growth are found in the right half of the plot, but those in the left half performed poorly under those conditions. The first component correlates positively with antioxidant enzyme activity, photosynthetic pigments, proline, shoot and leaf growth during stress, and root tissue density, whereas the second correlates negatively with shoot/root ratio, salt sensitivity index, and leaf tissue density. There is a positive correlation between stress-induced shoot and leaf growth and the activities of antioxidant enzymes, photosynthetic pigments, and proline content, while there is a negative correlation between stress-induced shoot and leaf growth and shoot/root, leaf tissue density, and SSI. (See Fig. 1B)

### Correlation analysis

**Figure 2:**
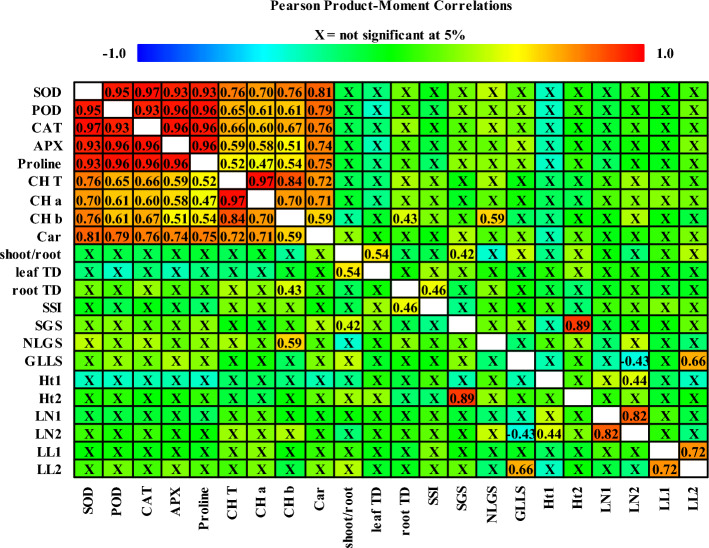
Correlation coefficients between the measured parameters of *C. spinosa*. *SOD* superoxide dismutase, *POD* peroxidase, *CAT* catalas, *APX* ascorbate peroxidase, *ChlT* total chlorophyll, *Chl a* chlorophyll a, *Chl b* chlorophyll b, *Car* carotenoid, *SSI* salt sensitivity index, *SGS* shoot growth during stress, *NLGS* number of new leaves growth during stress, *ILLS* increasing the length of largest leaf during stress, *LL1* length of largest leave before imposing stress, *LL2* length of largest leave at the end of trial. *LN1* number of leaves before imposing stress, *LN2* number of leaves at the end of trial, *Ht1* plant height before imposing stress, *Ht2* plant height at the end of trial.

Figure 2 depicts the result of the correlation analysis. The correlation analysis revealed a highly positive and significant relationship between different antioxidant enzymes and the proline content of caper. During drought and varied salt ratio stress, there is also a positive and significant correlation between photosynthetic pigments, antioxidant enzymes, and the proline content of caper. During stress, root tissue density and the number of new leaf growth revealed a positive and significant correlation with Chl *a*. The ratio of shoot/root has a positive and significant correlation with leaf tissue density. Furthermore, there is a significant positive correlation between SSI and root tissue density (Fig. 2).

### Path analysis

**Figure 3:**
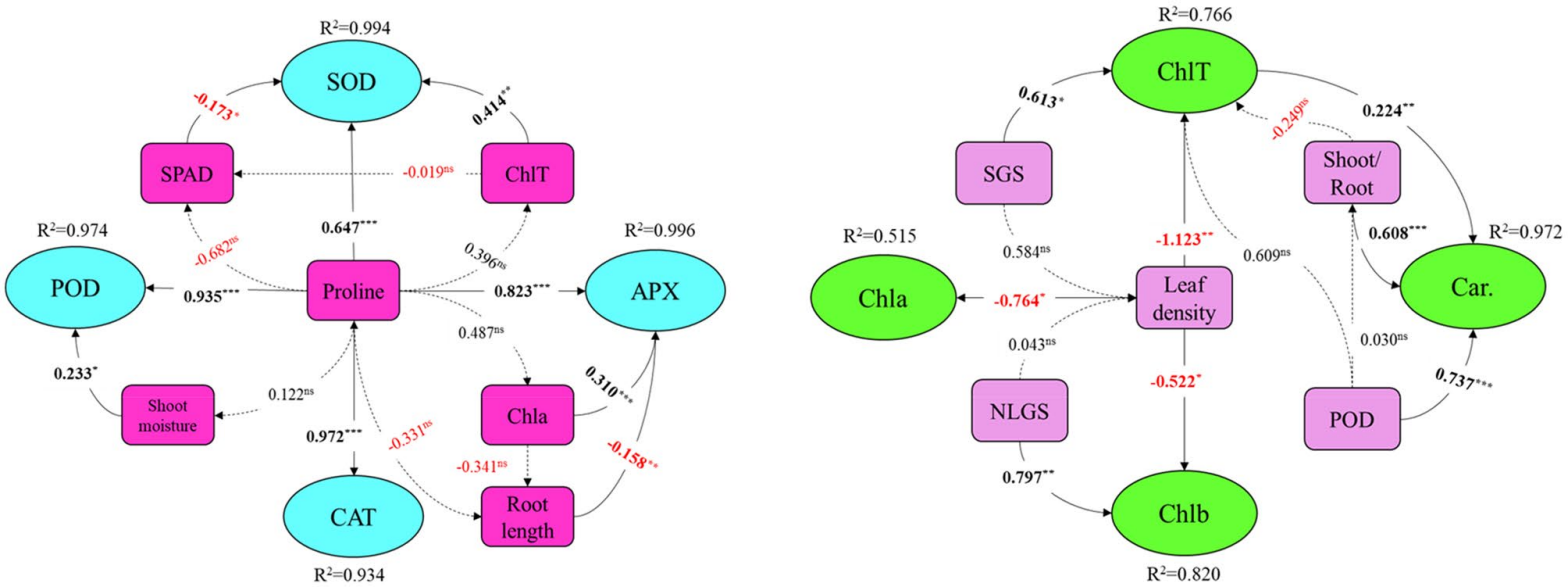
Path diagram showing the direct and indirect effect of different plant attributes on antioxidant enzymes activity and photosynthetic pigments of caper. *SOD* superoxide dismutase, *POD* peroxidase, *CAT* catalas, *APX* ascorbate peroxidase, *ChlT* total chlorophyll, *Chl a* chlorophyll a, *Chl b* chlorophyll b, *Car* carotenoid, *SPAD* chlorophyll index, *SGS* shoot growth during stress, *NLGS* number of new leaves growth during stress. Numerical values indicate *β* path coefficients between variables. Solid arrow: direct effect; Dashed arrow: indirect effect; Red values: negative beta value; Black values: positive beta value; ***, ** and * are significant at 0.001, 0.01 and 0.05 level of probability, respectively.

The path diagram was used to determine the relationship between morphophysiological parameters and the direct and indirect effects on photosynthetic pigments and antioxidant enzyme activity. It was put together using sequential multiple regression models (Fig. 3). ChlT and proline have a significant (*p* < *0.01* and *0.001*, respectively) and a positive effect on SOD, whereas the chlorophyll index (SPAD) has a significant (*p* < *0.01*) negative effect on it. Root length has a significant (*p* < *0.01*) negative effect on APX, while proline and chlorophyll have a significant (*p* < *0.001*) positive effect. Proline also has a significant (*p* < *0.001*) positive effect on CAT. In the case of POD, proline and shoot moisture have significant positive effects (*p* < *0.001* and 0.05, respectively) on enzyme activity. The path coefficient shows that proline has a higher effect on antioxidant enzyme activity than other parameters. Proline also has an indirect effect on antioxidant enzyme activity via other parameters that are statistically insignificant (Fig. 3).

In terms of photosynthetic pigments, leaf tissue density has a significant (*p* < *0.01*) negative effect on ChlT, while SGS has a significant (*p* < *0.05*) positive effect, and shoot/root has a non-significant negative effect. Furthermore, leaf tissue density had a negative significant (p < 0.05) effect on Chl *a* and *b*, but NLGS had a significant (*p* < *0.01*) and positive effect on Chl *b.* The effects of POD (*p* < *0.001*), shoot/root (*p* < *0.001*), and ChlT (*p* < *0.01*) on carotenoids were significant. The path coefficient shows that POD had a greater effect on carotenoids than other parameters (Fig. 3).

## Discussion

The effect of different CaCl_2_:NaCl salt ratios on the antioxidant defense system and morphophysiological parameters of caper during drought stress was investigated in this study. Zhang et al. demonstrated a significant enhancement in leaf tissue density in *Stellaria dichotoma* under water stress, but the current study found a significant decrease in caper leaf tissue density with 75% FC. The enhancement of leaf tissue density is primarily related to the decreasing size of leaves under water stress^31^. The opposite result of the current study might be due to the different ecological needs of the caper. Because caper has a high resistance to drought stress, mild water stress, such as 75% FC, not only did not impair the size of the leaves but actually enhanced leaf growth, and as a result, the leaf tissue decreased under drought stress. Furthermore, a significant enhancement in the largest leaf size of C. *spinosa* was observed at 75% FC while no changes in other morphological traits (such as root/shoot, SGS, and root tissue density) were observed, confirming that this plant is well adapted to mild drought stress (75% FC) and performs better under this condition. Most importantly, the result of the PCA analysis also supports the last finding. Sadeghi and Rostami^4^ demonstrated in a similar study that caper could tolerate drought stress (75% FC) with no noticeable changes in biochemical and physiological parameters.

In the current study, different CaCl_2_:NaCl salt ratios were employed to compare the effect of CaCl_2_ on caper under similar EC (8 dS/m). Based on the results, the ratio of 1:3 (CaCl_2_:NaCl) generated the highest shoot growth and number of new leaves growth during stress (NLGS), indicating that caper performed better at higher NaCl concentrations. This finding was further confirmed by PCA analysis, which revealed that caper performed better with 75% FC-1:3 CaCl_2_:NaCl. Xi et al. recently proposed that NaCl treatment significantly enhanced the plant biomass of *Zygophyllum xanthoxylum* by sustaining efficient photosynthesis while the stomata aperture was minimized^32^. Remarkably, further analysis of the current study confirms the above speculation, as the highest content of total chlorophyll and chlorophyll *a* were also identified in treatments with the highest levels of shoot and leaf growth.

Based on the results of the present study, the content of chlorophyll *a*, total chlorophyll, and carotenoid increased under the water stress. Drought reduces leaf area; thus cells are smaller than under normal conditions. However, the number of thylakoid and pigment apparatus stays unchanged, therefore the number of pigments per leaf area increases^33^. Based on the result of the present study, the highest content of chlorophyll *a*, *b,* and the total was found at 75% FC and the salt ratio of 1:3 to 1:5 (CaCl_2_:NaCl). Increasing chlorophyll content under salt stress was also observed by other researchers regarding *Citrus* × *aurantium*^34^, *Glycyrrhiza glabra*^35^ and *Olea europaea*^36^.

Based on the path analysis, leaf tissue density had a highly significant negative effect on chlorophylls (*a*, *b,* and total). This may be due to increases in CO_2_ diffusion caused by lower leaf tissue density which increases the photosynthesis efficiency^37,38^.

In terms of carotenoid content, the path diagram demonstrated that POD activity and shoot/root had a direct and significant positive effect. Furthermore, POD had an indirect effect on carotenoids via the shoot/root ratio. The latter results show that the main site of POD synthesis (under saline and drought stress) is most likely in leaves rather than roots, and that POD has a protective effect along the carotenoids against ROS (reactive oxygen species). Furthermore, carotenoids demonstrated a highly significant positive correlation with the activity of four antioxidant enzymes. Prior research has found a highly significant positive correlation between carotenoid and SOD, CAT, and APX in wheat leaf, which is consistent with the current results. They also introduced carotenoid as one of the most effective antioxidants to scavenge ROS among nine major evaluated antioxidants^24,39,40^. As a result, in addition to playing a photosynthetic role, carotenoids may operate as a non-enzymatic antioxidant for detoxifying oxygen radicals, and their level significantly enhances under drought and salinity stresses in the current study.

Based on the results of the present study, high NaCl concentration and drought stress conditions resulted in enhancing free proline content, which is in line with those previously observed in *Beta vulgaris* and *B. maritima*^41^, *Oryza sativa*^42^, and *Vicia faba* L.^43^. Organic solutes, such as proline, appear to play an important role in minimizing environmental stress by enhancing antioxidant enzyme activity and in maintaining water homeostasis in cells by decreasing osmotic potential in the cytoplasm^44^. The results of the path analysis and stepwise regression model equations also identified proline as the most important regressor in the activity of four antioxidant enzymes. The highest proline content and four antioxidant enzymes activity were seen at 75% FC and a salt ratio of 1:3 to 1:5. (CaCl_2_:NaCl). Several studies have found that higher proline accumulation is a crucial factor in resistance to environmental stresses^17,19,45^.

During environmental and biotic stress, the rate of ROS (reactive oxygen species) generation increases. ROS are inherently reactive molecules that can interact with various molecules and metabolites such as DNA, pigments, lipids, proteins, and other key cellular molecules, resulting in a series of damaging processes^46^. It is believed that antioxidant enzymes like SOD, APX, POD, and CAT decrease the level of ROS (such as hydrogen peroxide and superoxide) in plants^47^. The SOD, which is considered one of the most important enzymes used for reducing oxidative stress in the plant defense system, is responsible for catalyzing the dismutation of O_2_^−^ to molecular oxygen and hydrogen peroxide^48^. SOD induction in plant cells under various stressful environments reflects its role in plant defense mechanisms^4^. According to the path analysis, total chlorophyll and chlorophyll index, in addition to proline, had a significant effect on SOD activity, and plants with higher chlorophyll content demonstrated higher SOD activity which is resulted in better performance under stress conditions. This result is consistent with those observed in *Panicum virgatum* L.^49^, *Hordeum vulgare*^50^, *Raphanus sativus*^51^, and *Ailanthus altissima*^45^.

Catalase is another antioxidant enzyme that plays an important role in plant defense against oxidative stress. It catalyzes a redox reaction that results in the dismutation of hydrogen peroxide to produce oxygen and water^17,24^.

APX is the primary enzyme in plant cells for scavenging hydrogen peroxide in the chloroplast and cytosol, catalyzing ascorbate oxidation by hydrogen peroxide and producing monodehydroascorbate radical (MDA)^28^. Based on the results of path analysis from the current study, chlorophyll *a* and root length showed a significant positive and negative effect on APX activity, respectively. Moreover, there was a significant positive correlation between APX and chlorophyll *a*. These results are consistent with those reported by Ali et al., who observed a positive correlation between APX and chlorophyll content in wheat lines^52^. About the negative effect of root length on APX activity, to the best of the author's knowledge, no studies have been published that explore the possible relationship between APX and root length. Although, it has been reported that APX activity was significantly increased in the root of barley in saline conditions^42^. The latter results may be attributable to the site of APX synthesis, and it is likely that the main site of APX synthesis in caper under saline conditions is in roots rather than shoots. According to Hebelstrup and Moller^28^, plant tolerance to salt and water deficit improves by overexpressing APX in tobacco chloroplasts. Based on the obtained data, the salt sensitivity index of caper plants exposed to CaCl_2_ was less than NaCl. Similarly, it has been reported that CaCl_2_ had a less toxic effect on *Acer saccharinum* L. than sodium chloride^12^.

Regarding the predictive model equations, the negligible difference between measured and predicted values as well as the high regression coefficient (R^2^) indicates regression models worked well and can be utilized in the prediction of antioxidant enzymes activity and content of photosynthetic pigments of *C. spinosa* under drought and saline condition. Previous studies had employed regression models to predict plant response based on morphophysiological and biochemical parameters. Wang et al. employed regression models to predict antioxidant enzyme activity and MDA content in *Panicum virgatum* L. leaves and roots under NaCl-salinity stress^53^. Recently, regression modeling implemented to predict the growth of *Morus* Spp. genotypes using important morpho-metric traits and gas exchange parameters^54^. Unfortunately, there was no study at the time of writing this research on the possible relationships between antioxidant enzyme activities and pigment content with morphophysiological and biochemical plant parameters in drought and saline conditions.

The current study used predictive models and path analysis to highlight the critical role of proline content in the activity of four antioxidant enzymes. This finding can significantly minimize the high costs of measuring antioxidant enzymes in similar studies, and it is possible to estimate (with 99% confidence) the activity of those enzymes by quantifying proline content. Nonetheless, the models' localization may require additional testing, despite the fact that they are statistically significant in this study. Putti et al.,^51^ applied fuzzy modeling of salinity effects on radish yield and they verified that modelling will help to perform simulations capable of inferring points that were not experimentally determined. They could predict the yield of radish under different salinity treatment.

For the execution of predictive models in this study, the unit of proline should be g/gr Fw, and the activity of antioxidant enzymes should be put into the models as u/mg protein. The proline content of the leaves was the main variable in all antioxidant enzymes models. According to the path analysis, proline content accounts for 64.7% (SOD) to 97.2% (CAT) of the variance in the activity of four antioxidant enzymes with a confidence level of 99.9%. In fact, other variables in the predictive models of CAT, APX, and POD are insignificant, and the activity of those three enzymes can be estimated simply by quantifying proline content. The most influential variable in the execution of chlorophylls predictive models is leaf tissue density, while other variables have a negligible role in total variance of chlorophyll. According to the beta values obtained through path analysis, the most influential variables in the carotenoid model are POD activity and the shoot/root ratio, while the role of total chlorophyll is negligible in this model.

Recently, Singh et al.^55^ used PCA to discern shared and contrasting eco-physiological responses to salinity stress of *Ziziphus* rootstocks and budded trees. They discovered that PCA is extremely efficient at identifying rootstock- and salinity-specific effects in data. Likewise, in the current study PCA successfully discerned different salt ratio- and drought-specific effects in data, and treatments with better growth indices are easily recognizable.

## Conclusion

Drought and salinity stress have become big problem in many parts of the world today. Predicting the response of different plants under these conditions is quite useful and can be used to other plants as well as the advancement of research in this field. The results of the current study indicated that *C. spinosa* had a better performance under mild drought stress (75%FC) and a salt ratio of 1:3 (CaCl_2_:NaCl) rather than in non-stress conditions. According to PCA analysis, treatments with higher performance had higher content of photosynthetic pigments, proline, and antioxidant enzyme activity. Under drought and saline conditions, the developed regression models accurately predicted the content of proline, pigments, and antioxidant enzyme activity of caper. The values of *R*^*2*^ ranged from 0.893 to 0.998 approving high linear fitness of the regression models (except for Chl *a*, 0.584). The correlation analysis revealed a highly positive and significant correlation between several antioxidant enzymes and the proline content of caper. The path diagram demonstrated that the content of proline was the most effective independent variable on antioxidant enzyme activity. It was also discovered that leaf density has a significant negative impact on chlorophylls (*a, b* and total).

The results of this study would be a useful guide for caper producers as well as plant ecophysiological researchers for more investigations on the possible connection between the plant antioxidant defense system with photosynthesis pigments.

## Supplementary Information

Supplementary Tables.

## Data Availability

The datasets used and/or analyzed during the current study are available from the corresponding author upon reasonable request.
