# Supplementary material for: A comprehensive model for predicting the development of defense system of Capparis spinosa L.: a novel approach to assess the physiological indices
Source: Sci Rep. 2023 Jul 31;13:12413. doi: 10.1038/s41598-023-39683-5 (PMC10390471; doi:10.1038/s41598-023-39683-5)
Supplement: Supplementary file 1 — Supplementary Tables. [file 41598_2023_39683_MOESM1_ESM.docx]

| Table S1. Some of physiochemical properties of the pot soil. | | | | | | | | | |
| --- | --- | --- | --- | --- | --- | --- | --- | --- | --- |
| OC  (%) | pH | Sand  (%) | Silt  (%) | Clay  (%) | Soil texture | EC  (dSm^-1^) | P  (mg kg^-1^) | K  (mg kg^-1^) | Total N  (%) |
| 0.78 | 7.50 | 7.6 | 67.1 | 25.3 | Silty loam | 0.61 | 15.5 | 456 | 0.07 |

Table S2. Analyses of variance of the measured factors of caper.

| Source | df | Mean square | | | | | | | |
| --- | --- | --- | --- | --- | --- | --- | --- | --- | --- |
|  |  | Shoot/Root | SSI | SGS | NLGS | ILLS | LDensity | RDensity |  |
| Drought | 1 | 0.01^ns^ | 0.04^ns^ | 5.51^ns^ | 0.041^ns^ | 0.95* | 0.02* | 0.001^ns^ |  |
| Salt Ratio | 3 | 1.07** | 0.25** | 57.23^ns^ | 13.82** | 0.14^ns^ | 0.003^ns^ | 0.006^ns^ |  |
| Drought × Salt Ratio | 3 | 1.26** | 0.05^ns^ | 70.79* | 0.82^ns^ | 0.07^ns^ | 0.02** | 0.003^ns^ |  |
| Error | 16 | 0.091 | 0.03 | 20.24 | 2.375 | 0.13 | .004 | .003 |  |
| C.V | - | 41 | 36 | 47 | 48 | 42 | 43 | 26 |  |

| Source | df | Mean square | | | | | | | | | |
| --- | --- | --- | --- | --- | --- | --- | --- | --- | --- | --- | --- |
|  |  | SOD | POD | CAT | APX | proline | cht | cha | chb | car |  |
| Drought | 1 | 112.18** | 147.44** | 108.03** | 102.84** | 31.87** | 19.77^ns^ | 14.02^ns^ | 0.49^ns^ | 5.72** |  |
| Salt Ratio | 3 | 7.83^ns^ | 8.86^ns^ | 4.41^ns^ | 2.26^ns^ | 1.69^ns^ | 5.70^ns^ | 5.48^ns^ | 1.445^ns^ | 1.35^ns^ |  |
| Drought × Salt Ratio | 3 | 3.18^ns^ | 3.09^ns^ | 1.06^ns^ | 1.22^ns^ | 0.21^ns^ | 27.44^ns^ | 17.00* | 1.35^ns^ | 0.24^ns^ |  |
| Error | 16 | 6.848 | 5.575 | 4.332 | 1.845 | 1.041 | 9.369 | 4.485 | 1.103 | .595 |  |
| C.V | - | 33 | 37 | 39 | 41 | 42 | 23 | 24 | 27 | 25 |  |

Table S3. The list of measured variables which were used for path analysis and stepwise regression models.

| No. | Measured variables (un shown data) |
| --- | --- |
| 1 | The length of first node |
| 2 | Chlorophyll index (SPAD) |
| 3 | Shoot fresh weight |
| 4 | Shoot dry weight |
| 5 | Shoot turgid weight |
| 6 | Shoot moisture |
| 7 | Shoot relative water content |
| 8 | Shoot water saturated deficit |
| 9 | Shoot length |
| 10 | Root fresh weight |
| 11 | Root dry weight |
| 12 | Root turgid weight |
| 13 | Root moisture |
| 14 | Root relative water content |
| 15 | Root water saturated deficit |
| 16 | Root length |
| 17 | Total fresh weight |
| 18 | Total dry weight |

Table S4. The eigenvectors, proportion of variation and cumulative contributions of variance accounted for each principal component that was estimated from 18 variables of caper seedlings treated under different salt ratio and water stress treatments.

| **Variable** | **PC1** | **PC2** | **PC3** | **PC4** | **PC5** | **PC6** | **PC7** | **PC8** | **PC9** | **PC10** |
| --- | --- | --- | --- | --- | --- | --- | --- | --- | --- | --- |
| SOD | 0.356 | -0.014 | -0.077 | 0.021 | -0.060 | -0.029 | 0.142 | 0.268 | -0.004 | 0.315 |
| POD | 0.346 | 0.035 | -0.044 | 0.110 | -0.073 | -0.305 | -0.156 | -0.026 | -0.410 | -0.140 |
| CAT | 0.343 | -0.022 | -0.126 | 0.183 | -0.008 | -0.025 | -0.166 | 0.026 | 0.542 | 0.077 |
| APX | 0.346 | 0.048 | -0.059 | 0.193 | -0.008 | -0.070 | -0.249 | -0.135 | 0.175 | -0.010 |
| Proline | 0.324 | 0.024 | -0.175 | 0.232 | -0.109 | -0.243 | -0.039 | -0.131 | -0.287 | -0.151 |
| CH T | 0.273 | -0.023 | 0.278 | -0.364 | -0.071 | 0.277 | -0.113 | -0.227 | -0.115 | -0.172 |
| CH a | 0.257 | 0.063 | 0.329 | -0.301 | -0.096 | 0.345 | -0.223 | -0.159 | 0.116 | 0.128 |
| CH b | 0.221 | -0.276 | 0.024 | -0.421 | 0.030 | -0.030 | 0.262 | 0.138 | -0.047 | -0.207 |
| Car | 0.278 | 0.251 | -0.146 | -0.119 | -0.163 | -0.045 | 0.042 | -0.088 | 0.193 | -0.019 |
| shoot/root | -0.066 | 0.379 | -0.270 | -0.242 | -0.025 | 0.024 | -0.034 | -0.078 | -0.388 | 0.239 |
| leaf TD | -0.228 | 0.083 | -0.460 | -0.070 | -0.088 | 0.098 | -0.077 | -0.314 | 0.326 | -0.340 |
| ROOT TD | 0.071 | -0.278 | -0.020 | -0.033 | 0.787 | -0.137 | -0.116 | -0.417 | -0.031 | 0.183 |
| SSI | -0.134 | 0.387 | 0.099 | -0.113 | 0.231 | -0.079 | -0.671 | 0.358 | -0.021 | -0.114 |
| SGS | 0.051 | 0.139 | -0.482 | -0.448 | 0.051 | -0.076 | 0.070 | -0.095 | 0.020 | 0.231 |
| NLGS | 0.088 | -0.362 | -0.268 | -0.266 | 0.137 | -0.065 | -0.138 | 0.527 | 0.063 | -0.263 |
| GLLS | 0.191 | 0.282 | -0.126 | 0.215 | 0.358 | 0.479 | 0.247 | 0.300 | -0.012 | 0.177 |
| LL1 | -0.029 | 0.305 | 0.336 | -0.229 | 0.070 | -0.604 | 0.254 | 0.056 | 0.310 | 0.141 |
| LL2 | 0.134 | 0.389 | 0.085 | 0.044 | 0.320 | 0.046 | 0.334 | -0.042 | 0.005 | -0.608 |
| Eigenvalue | 7.695 | 4.847 | 2.588 | 1.391 | 0.928 | 0.456 | 0.092 | 0.000 | 0.000 | 0.000 |
| Proportion | 0.428 | 0.269 | 0.144 | 0.077 | 0.052 | 0.025 | 0.005 | 0.000 | 0.000 | 0.000 |
| Cumulative | 0.428 | 0.697 | 0.841 | 0.918 | 0.970 | 0.995 | 1.000 | 1.000 | 1.000 | 1.000 |
|  |  |  |  |  |  |  |  |  |  |  |
|  |  |  |  |  |  |  |  |  |  |  |
|  |  |  |  |  |  |  |  |  |  |  |
